# Multi-omics biomarkers for predicting resistance, hyperprogression, and immune-related toxicity during PD-1/PD-L1 therapy in lung cancer: a literature review

**DOI:** 10.3389/fimmu.2026.1780459

**Published:** 2026-05-08

**Authors:** Na Hu, Ziyue Wang, Ming Liu, Dong Guo, Chenxue Jiang, Yun Chen, Zhenshan Zhang, Menglin Bai, Bo Cheng, Jessica C. Hsu, Leilei Wu, Xiujie Sui

**Affiliations:** 1 Department of Radiotherapy, Yantaishan Hospital, Yantai, Shandong, China; 2 School of Nursing and Midwifery, University of Galway, Galway, Ireland; 3 Department of Radiation Oncology, Shanghai Pulmonary Hospital, School of Medicine, Tongji University, Shanghai, China; 4 Department of Radiation Oncology, Weifang People’s Hospital, Shandong Second Medical University, Weifang, China; 5 Department of Thoracic Surgery, Shanghai Pulmonary Hospital, School of Medicine, Tongji University, Shanghai, China; 6 Department of Radiation Oncology, Qilu Hospital of Shandong University, Jinan, Shandong, China; 7 School of Health Sciences, Purdue University, West Lafayette, IN, United States

**Keywords:** biomarkers, immune-related adverse events, lung cancer, multi-omics, PD-1/PD-L1 inhibitors, resistance

## Abstract

Immune checkpoint inhibitors targeting programmed cell death protein 1 (PD-1) and its ligand programmed death-ligand 1 (PD-L1) have transformed the management of advanced lung cancer, yet most patients experience primary resistance, hyperprogressive disease (HPD), or clinically significant immune-related adverse events (irAEs). Multi-omics technologies now enable integrated interrogation of tumor, microenvironmental, host, and clinical determinants of these divergent outcomes. In this review, we first discuss the biological and clinical foundations of PD-1/PD-L1 blockade in non-small cell and small cell lung cancer, and summarize the spectrum of resistance, HPD, and irAEs observed in trials and real-world practice. We then describe multi-omics study frameworks that connect genomics, transcriptomics, epigenomics, proteomics, metabolomics, radiomics, and microbiome profiling with these outcome phenotypes. Building on this foundation, we synthesize evidence for composite biomarkers of primary and acquired resistance, delineate emerging multi-omics signatures of HPD, and examine host- and tumor-derived multi-omics correlates of organ-specific and systemic irAEs. We further propose an efficacy–risk quadrant framework to guide clinical decision-making when favorable efficacy predictors coexist with elevated risk of severe adverse outcomes, and outline a three-step approach for high-efficacy/high-risk patients: joint probability reporting, multi-omics guided mitigation, and dynamic reassessment. Finally, we evaluate translational strategies that integrate multi-omics scores into baseline risk stratification, dynamic monitoring with attention to technical challenges such as distinguishing true progression from ctDNA pseudoprogression, and biomarker-driven trial design, while assessing the evidence level and translational readiness of candidate assays from retrospective discovery to clinical implementation. A clinical case illustrates how multi-omics can link baseline risk stratification, regimen selection, and longitudinal monitoring into a coherent action plan, while acknowledging that artificial intelligence-driven models remain investigational and real-world application still relies on clinician judgment. Collectively, this review defines how integrated multi-omics biomarkers can be leveraged to predict resistance, HPD, and immune-related toxicity, and to refine patient selection and management during PD-1/PD-L1 therapy in lung cancer.

## 1 Introduction

Immune checkpoint inhibitors (ICIs) targeting programmed cell death protein 1 (PD-1) and its ligand programmed death-ligand 1 (PD-L1) have reshaped the treatment landscape of lung cancer, demonstrating clinical benefit across multiple disease stages in both non-small cell lung cancer (NSCLC) and small cell lung cancer (SCLC) (1–4). Despite these advances, durable responses remain limited to a subset of patients (5, 6), and substantial heterogeneity persists even among biomarker-selected populations (6, 7).

The cancer-immunity cycle provides a conceptual framework for understanding these heterogeneous outcomes (8, 9). This cycle comprises sequential steps including tumor antigen release, antigen presentation, T-cell priming, trafficking, infiltration, recognition, and killing, each of which can be disrupted by tumor-intrinsic, microenvironmental, or host-related factors. Clinical responses to PD-1/PD-L1 blockade therefore depend not only on checkpoint inhibition at the effector phase, but also on the integrity of upstream immune activation processes across the cycle (8, 9).

Among patients receiving PD-1/PD-L1 blockade in lung cancer, clinically unfavorable outcomes include resistance, hyperprogressive disease (HPD), and immune-related adverse events (irAEs). These outcomes are jointly shaped by therapeutic efficacy and treatment-related toxicity, which, despite partially overlapping biological mechanisms, reflect distinct clinical priorities, namely enhancing antitumor efficacy and minimizing treatment-related harm (10, 11). Accordingly, they are best interpreted along two complementary dimensions, efficacy and safety. Within the efficacy dimension, patients may achieve durable clinical benefit, exhibit primary or acquired resistance, or, in a subset of cases, develop HPD, which represents an aggressive form of treatment failure (5, 6, 12, 13). In parallel, irAEs arise along the safety dimension, reflecting treatment-associated toxicity rather than therapeutic efficacy (14, 15).

While resistance and HPD are often described as separate clinical entities, emerging evidence suggests that they share common biological underpinnings (16–18). Both are driven by tumor-intrinsic programs, suppressive tumor microenvironmental states, and systemic permissive conditions that impair effective antitumor immunity. In this context, primary resistance reflects ineffective or excluded immune responses present before or early during therapy, whereas acquired resistance reflects adaptive reprogramming or evolutionary escape after an initial period of benefit (5, 12, 13). HPD may represent a more dynamic and accelerated failure state driven by dysregulated tumor–immune interactions (17, 18). By contrast, irAEs arise through distinct mechanisms involving excessive or misdirected immune activation, influenced by host-specific and systemic factors (14, 15, 19). Together, these observations support a framework in which resistance and HPD lie along a continuum of tumor-dominant therapeutic failure, whereas irAEs represent immune-dominant dysregulation.

These considerations underscore the limitations of single-parameter biomarkers such as PD-L1 expression or tumor mutational burden (TMB), each of which captures only a restricted aspect of the cancer-immunity cycle (8, 9, 20). Clinical outcomes instead emerge from coordinated perturbations across multiple biological layers, including tumor genomics, transcriptional programs, proteostasis networks, metabolic states, and host immune regulation (20–22). Multi-omics technologies provide an opportunity to systematically capture these multi-layered determinants (22). By integrating genomics, transcriptomics, epigenomics, proteomics, metabolomics, microbiome profiling, and imaging-derived features, multi-omics approaches enable a more comprehensive characterization of tumor–immune–host interactions. Importantly, such approaches not only improve predictive performance but also offer mechanistic insights into how tumor-dominant failure and immune-dominant toxicity arise from shared yet context-dependent biological processes.

In this review, we synthesize current evidence on multi-omics biomarkers in lung cancer immunotherapy and propose an integrated conceptual framework linking molecular features to clinically relevant phenotypes. We focus on tumor-dominant failure states encompassing resistance and HPD, and on immune-dominant toxicity represented by irAEs. We further discuss how these insights can be translated into clinically actionable strategies for patient stratification, dynamic monitoring, and rational trial design in the context of PD-1/PD-L1 blockade.

## 2 Biological and clinical background of PD-1/PD-L1 blockade in lung cancer

PD-1 is an inhibitory receptor induced on activated CD4^+^ and CD8^+^ T cells, B cells, natural killer cells, and some myeloid subsets, whereas its ligands PD-L1 and PD-L2 are expressed on antigen-presenting cells, endothelial cells, and many epithelial tumors including NSCLC (23, 24). Within the cancer-immunity cycle, PD-1/PD-L1 blockade primarily acts at the recognition and killing phases of the antitumor response, but clinical efficacy also depends on intact upstream antigen release, presentation, T-cell priming, trafficking, and infiltration (8, 9). Engagement of PD-1 by PD-L1 recruits SHP2, which preferentially dephosphorylates CD28 and dampens proximal TCR signaling, thereby attenuating PI3K-AKT and RAS-MAPK pathways (24). This results in reduced IL-2, TNF, and IFN-γ production, impaired proliferation, and metabolic reprogramming characterized by suppressed glycolysis and increased fatty-acid oxidation, a hallmark of T-cell exhaustion (25, 26). Sustained signaling through this axis in the tumor microenvironment stabilizes a dysfunctional effector phenotype, limits clonal expansion of tumor-reactive T cells, and permits survival of PD-L1-high lung cancer clones with diminished immunogenicity.

The cancer-immunity cycle provides a systematic framework to understand how tumor-intrinsic and microenvironmental factors converge to determine clinical outcomes (8, 9). At each step of the cycle, distinct determinants come into play. Antigen release and presentation are shaped by TMB and HLA genotype as well as dendritic cell activation (23, 27). T-cell priming and trafficking are modulated by chemokine gradients and stromal barriers influenced by tumor-derived signals such as TGF-β (6, 27, 28). Infiltration and recognition require coordinated function of endothelial adhesion molecules and MHC-peptide complexes, abnormalities of which have been linked to both resistance and HPD (6, 16, 29). The killing phase, while directly targeted by PD-1/PD-L1 inhibitors, is constrained by microenvironmental suppressors including alternative immune checkpoints, myeloid-derived suppressor cells (MDSCs), and T-cell exhaustion programs (24). Thus, multi-omics biomarkers can be mapped onto these sequential steps, yet their interpretation must account for the intertwined nature of tumor-intrinsic and microenvironmental drivers.

The lung cancer immune microenvironment is heterogeneous. Tobacco-associated NSCLC typically exhibits high TMB and abundant neoantigens, favoring baseline CD8^+^ T-cell infiltration but imposing strong selective pressure for adaptive PD-L1 upregulation on tumor and myeloid cells (30). In contrast, oncogene-addicted NSCLC driven by *EGFR*, *ALK* or other actionable alterations often displays lower mutational burden, altered interferon signaling, and sparse effector T-cell infiltration, with PD-L1 expression frequently uncoupled from inflamed gene-expression signatures and predicting limited benefit from PD-1/PD-L1 blockade (31). SCLC combines high genomic instability with profound immunosuppression, including abundant myeloid cells and regulatory lymphocytes, which may explain the modest incremental survival gains observed when PD-L1 inhibitors are added to platinum-etoposide chemotherapy (3, 32). As shown in **Table 1**, tumor-intrinsic alterations, microenvironmental composition, systemic host factors, and clinical characteristics jointly determine the functional state of the PD-1/PD-L1 axis and clinical responsiveness to checkpoint blockade in lung cancer.

**Table 1:** Key biological and clinical determinants of PD-1/PD-L1 blockade in lung cancer.

Level	Representative features	Examples in lung cancer	Implications for PD-1/PD-L1 therapy
Tumor-intrinsic	Somatic mutations, copy-number changes, oncogenic drivers, antigen-presentation machinery, transcriptomic immune signatures	High tumor mutational burden in smoking-related NSCLC; EGFR/ALK-driven tumors with low mutational burden; loss or downregulation of HLA class I and β2-microglobulin; interferon-response gene programs	Influence neoantigen load, basal PD-L1 expression, and susceptibility to T-cell recognition; shape probability of primary and acquired resistance despite PD-L1 positivity
Tumor microenvironment	Density, phenotype, and spatial distribution of CD8^+^ T cells, regulatory T cells, myeloid-derived suppressor cells, tumor-associated macrophages, stromal architecture	“Inflamed” versus “immune-excluded” or “immune-desert” NSCLC; myeloid-dominant infiltrates; desmoplastic or hypoxic niches	Modulate effective engagement of PD-1/PD-L1 axis, depth of response, and propensity for HPD under checkpoint inhibition
Systemic and host immunity	Peripheral immune-cell subsets, soluble checkpoint ligands, cytokines, chemokines, microbiome, germline variants	Baseline PD-1^+^ or TIGIT^+^ exhausted-like CD8^+^ T cells; soluble PD-L1; chronic inflammatory comorbidities; microbiome diversity	Affect systemic pharmacodynamic response, risk of immune-related toxicity, and durability of benefit beyond the primary tumor site
Clinical context	Histology, disease stage, prior treatments, performance status, organ function, coexisting autoimmune disease	Advanced versus locally advanced NSCLC; extensive-stage small-cell lung cancer; prior thoracic irradiation; pre-existing interstitial lung disease or autoimmunity	Constrains indication, dosing, and monitoring strategies; interacts with biological factors to determine net benefit–risk profile of PD-1/PD-L1 blockade

Randomized phase III trials have established PD-1/PD-L1 inhibitors as standard therapy across multiple stages of NSCLC. In the first landmark trial, KEYNOTE-024, pembrolizumab monotherapy significantly improved overall and progression-free survival in metastatic NSCLC with high PD-L1 expression, leading to its approval as first-line treatment (1), and further demonstrated survival benefits in broader PD-L1-positive cohorts compared to chemotherapy alone (33). The two subsequent trials, KEYNOTE-189 (34) and KEYNOTE-407 (35), established first-line immunochemotherapy combinations as a standard treatment, demonstrating survival benefits irrespective of PD-L1 expression. However, effective biomarkers remain crucial for patient stratification and tailoring treatment strategies. Beyond immunochemotherapy combinations, diverse combination strategies including dual immune checkpoint inhibition, regimens integrating anti-angiogenic therapies, the rapid development of bispecific antibodies, and the incorporation of radiotherapy have collectively broadened treatment paradigms and increased therapeutic options for patients with various stages of NSCLC (7, 27).

Across these indications, only a minority of unselected patients achieve long-term tumor control, and outcome heterogeneity is pronounced even among those with apparently favorable biomarkers such as high PD-L1 expression or elevated TMB. Retrospective analyses in NSCLC show that a substantial fraction of PD-L1-high tumors exhibit primary resistance with early progressive disease (36), whereas others develop acquired resistance after an initial response, accompanied by dynamic modulation of PD-L1, loss of antigen-presentation components, or remodeling of myeloid and stromal compartments (37, 38). A subset of patients develop HPD, defined by acceleration of tumor growth kinetics and early clinical deterioration shortly after starting PD-1/PD-L1 blockade; case series and cohort studies in lung cancer suggest clinically relevant incidence and uniformly poor prognosis, but mechanistic underpinnings and predictive factors remain incompletely understood (39, 40).

Beyond efficacy, irAEs represent an additional source of clinical heterogeneity. PD-1/PD-L1 inhibitors can induce organ-specific or systemic auto-inflammatory toxicities affecting skin, endocrine organs, lung, gastrointestinal tract, heart, and the nervous system, with overall incidence generally lower than with CTLA-4 blockade but substantial in real-world NSCLC cohorts (19). Observational studies in advanced NSCLC indicate that mild to moderate irAEs correlate with improved overall survival, whereas severe irAEs associate with worse outcomes and frequent treatment discontinuation, suggesting that toxicity and efficacy share overlapping but not identical immunologic determinants (11). Collectively, these biological and clinical observations support the view that PD-1/PD-L1 blockade outcomes in lung cancer arise from multi-layer perturbations extending from tumor genomics and transcriptional programs to microenvironmental composition, circulating immune and soluble factors, and germline or environmental host traits. It should be noted, however, that the vast majority of multi-omics biomarker studies have been conducted in NSCLC, reflecting its higher prevalence and broader therapeutic indications. Where SCLC-specific data exist, they are highlighted; otherwise, findings are reported as applicable to lung cancer generally, with the caveat that direct extrapolation to SCLC requires prospective validation.

## 3 Multi-omics study frameworks

Multi-omics study frameworks for PD-1/PD-L1 therapy in lung cancer connect heterogeneous molecular measurements with clinically defined outcomes, including resistance, HPD, and immune-related adverse events. Tumor, microenvironment, and host are conceptualized as interacting biological systems (20, 28). Coordinated acquisition of tumor genomics and epigenomics, bulk and single-cell transcriptomics, quantitative proteomics, metabolomics, microbiome profiling, and imaging-derived features is coupled to detailed annotation of treatment exposure, response kinetics, and toxicity (22, 41). As shown in **Table 2**, multi-layer datasets are typically organized by biological compartment, omics modality, and study design dimension, encompassing longitudinal sampling, discovery and validation cohorts, and both trial and real-world settings.

**Table 2:** Core design dimensions of multi-omics study frameworks for PD-1/PD-L1 therapy in lung cancer.

Axis	Key elements	Typical implementation in PD-1/PD-L1 lung cancer studies
Biological compartment	Tumor tissue; tumor microenvironment; systemic circulation; microbiome	Resected or biopsied primary and metastatic lesions; multiplex tissue profiling of immune and stromal cells; serial peripheral blood sampling; stool or airway specimens for microbial profiling.
Omics modality	Genomics and epigenomics; transcriptomics; proteomics and phosphoproteomics; metabolomics; microbiome; radiomics	Exome or targeted-panel sequencing and methylation assays; bulk and single-cell RNA sequencing; mass-spectrometry–based proteomics and metabolomics; 16S or shotgun metagenomics; CT- or PET-derived radiomic features.
Study design dimension	Sampling schedule; cohort structure; clinical endpoints	Baseline and on-treatment sampling around PD-1/PD-L1 initiation; discovery and external validation cohorts; prospective trial-embedded or retrospective real-world designs; standardised definitions of resistance, HPD, and immune-related toxicity.
Analytical strategy	Data integration and modelling	Early feature-level or late model-level integration; unsupervised clustering and dimensionality reduction; supervised machine-learning models for risk scores or response classifiers with calibration and independent validation.

### 3.1 Tissue biopsy-based approaches

Tumor-tissue-centered frameworks typically use resected or biopsied lung cancer samples linked to PD-1/PD-L1 treatment outcomes or surrogate endpoints such as immune infiltration and interferon signatures. Proteogenomic studies combining exome sequencing, transcriptomics, and deep proteomics in lung adenocarcinoma have delineated molecular subtypes with distinct immune-evasion programs and therapeutic vulnerabilities, providing a reference resource for immunotherapy biomarker discovery (42). Multi-omics workflows within NSCLC integrate tumor-intrinsic lesions with immune features by jointly analyzing transcriptomic, mutational, copy-number, and methylation data, thereby defining subgroups with differential checkpoint expression and inferred sensitivity to ICIs (43). Related analyses construct gene signatures that stratify patients by prognosis, immune infiltration, and predicted benefit from PD-1-directed therapy, with validation across independent cohorts (44). Pan-cancer models that merge TCGA multi-omics data with clinical trial outcomes show that composite scores integrating neoantigen load with immune and metabolic features outperform single biomarkers when predicting long-term survival after immunotherapy, including in lung cancer (45).

Tissue-based approaches offer unique advantages, including the ability to resolve histology, spatial immune architecture, stromal exclusion, cell–cell proximity, phosphoprotein states, and direct tumor–microenvironment interactions. Despite these strengths, they also have important limitations. Single-site sampling may fail to capture the full clonal and immune heterogeneity of disseminated lung cancer, as a single primary or metastatic biopsy often does not represent spatially distinct subclones or divergent immune infiltration patterns across lesions (46, 47). In addition, the procedural burden of invasive biopsies introduces risks of complications and limits patient acceptability, particularly for longitudinal sampling. Furthermore, limited repeatability and inherent temporal lag hinder the ability to track dynamic changes in tumor biology and the immune microenvironment during treatment, thereby constraining real-time monitoring of emerging resistance and pharmacodynamic responses.

### 3.2 Liquid biopsy-based approaches

Liquid biopsy-based approaches, primarily based on peripheral blood but also encompassing other biofluids such as urine and cerebrospinal fluid, provide a minimally invasive alternative that enables serial sampling and real-time monitoring of tumor dynamics. Peripheral-blood-based designs integrate baseline and on-treatment circulating tumor DNA (ctDNA), circulating immune cells, soluble proteins, metabolites, and extracellular vesicles with survival and toxicity endpoints, enabling development of blood-based gene and protein signatures that stratify response to ICIs in independent cohorts (46–49).

The advantages of blood-based assays include reduced sampling burden, suitability for serial measurements, whole-body disease tracking, and early pharmacodynamic monitoring. However, they provide less direct spatial context and can be compromised by low analyte abundance, clonal hematopoiesis, variable tumor shedding, and uncertainty regarding the anatomical source of the signal.

### 3.3 Imaging-based approaches

Imaging-based approaches provide non-invasive, spatially resolved information on tumor metabolic activity, heterogeneity, and treatment response. ¹^8^F-FDG PET parameters such as SUVmax, metabolic tumor volume (MTV), and total lesion glycolysis (TLG) reflect tumor glucose uptake and glycolytic activity, which correlate with tumor aggressiveness and immune microenvironment status (50, 51). Radiomics approaches extract high-dimensional features from CT, MRI, and PET images to capture intratumoral heterogeneity and predict response to immunotherapy (52). These imaging biomarkers complement molecular data by providing whole-tumor spatial information that cannot be captured by single-site biopsies.

### 3.4 Other biomarker sources

Beyond tissue, liquid, and imaging-based approaches, several other sources contribute to the multi-omics landscape of immunotherapy response. Microbiome-derived biomarkers have emerged as key modulators of systemic immune responses, with metagenomic and 16S rRNA sequencing of fecal samples identifying microbial signatures associated with response to PD-1/PD-L1 blockade, including enrichment of *Akkermansia muciniphila*, *Bifidobacterium species*, and other commensal bacteria (53–55). Host genetic biomarkers, including germline variants in human leukocyte antigen (HLA) genes and other immune-related loci, influence both treatment response and susceptibility to immune-related adverse events (56–59). Clinical biomarkers such as performance status, smoking history, comorbidities, and inflammatory indices (e.g., neutrophil-to-lymphocyte ratio) provide readily accessible but often underutilized predictors of outcome (30, 37, 38, 60).

### 3.5 Integration strategies and clinical translation

Translationally oriented multi-omics study frameworks embed provisions for biological interpretation and future implementation. The most informative approaches integrate multiple biomarker sources, pairing tissue-derived mechanistic resolution at baseline with longitudinal liquid-biopsy readouts during treatment, and incorporating imaging and clinical data to capture tumor heterogeneity, metabolic state, and host immune tone comprehensively (22, 41, 61).

A critical distinction in routine practice is between baseline tools and on-treatment monitoring tools. Baseline tools are most useful for pretreatment stratification, including estimating the probability of primary resistance, selecting first-line monotherapy versus combination therapy, and identifying patients with pre-existing toxicity vulnerability. These tools benefit from stable sampling windows and clearer treatment-decision thresholds. In contrast, on-treatment monitoring tools are better suited for detecting emerging pharmacodynamic response, adaptive resistance, acquired resistance, incipient HPD, and subclinical irAEs. Monitoring assays must prioritize rapid turnaround, repeatability, and resistance to confounding by intercurrent infection, corticosteroids, antibiotics, or variable ctDNA shedding (46, 47, 62–66). This distinction is essential for translating multi-omics findings into clinically actionable workflows.

Pathway analysis enables the integration of predictive signatures into established biological processes, including interferon–JAK/STAT signaling, antigen-processing machinery, stromal and myeloid programs, metabolic pathways, and microbiome-related signals, thereby prioritizing biomarkers that are both statistically robust and mechanistically coherent (43, 44). Beyond early and late integration, emerging systems-immunology approaches use Bayesian networks to represent conditional dependencies between genomic events, causal-inference frameworks to link alterations across transcript, protein, and metabolite layers, and graph-based learning to model cell states, pathways, and cross-omic interactions within a common relational structure (67–69). These approaches are especially attractive when the objective is not only classification but mechanistic explanation, namely, how clonal architecture propagates into immune exclusion, metabolic reprogramming, or toxicity-prone host states.

Recent reviews of lung cancer multi-omics emphasize rigorous pre-analytic standardization, transparent reporting of integration and modeling strategies, and prospective validation in uniformly treated PD-1/PD-L1 cohorts to enable clinical uptake (70). As these frameworks mature, they provide a basis for composite biomarkers that jointly estimate the probability of primary resistance, the risk of HPD, and the propensity for immune-related toxicity during PD-1/PD-L1 therapy.

## 4 Multi-omics biomarkers of resistance to PD-1/PD-L1 inhibitors in lung cancer

**Figure 1:**
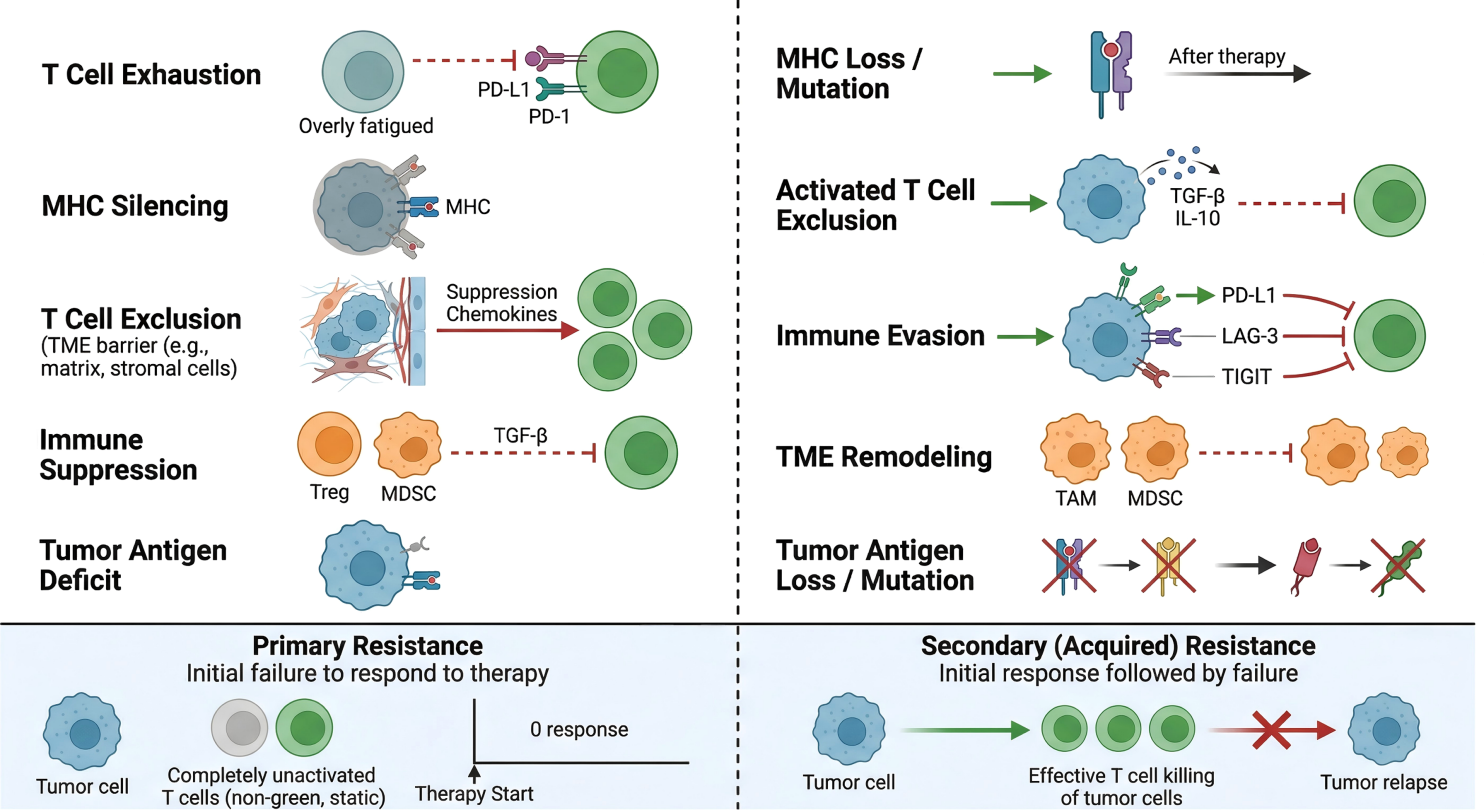
Clinical phenotypes of resistance to PD-1/PD-L1 blockade. Resistance is classified into primary resistance (no objective response or progression within 6 months) and acquired resistance (progression after initial benefit ≥6 months). The two phenotypes differ in onset timing, clinical trajectory, and underlying drivers.

Resistance to PD-1/PD-L1 blockade is not a single entity but encompasses two clinically distinct phenotypes, namely primary resistance and acquired resistance, observed across both monotherapy and combination treatment settings (5, 12, 13). Primary resistance refers to the failure to achieve any objective response or the occurrence of early progressive disease within the first several months of therapy, typically within 6 months, despite adequate exposure to checkpoint inhibitors. This phenotype reflects pre-existing tumor-intrinsic, microenvironmental, or host features that render the cancer inherently unresponsive from the outset. In contrast, acquired resistance occurs in patients who initially derive clinical benefit, including partial or complete response or stable disease lasting at least 6 months, but subsequently experience disease progression. This phenotype arises from dynamic reprogramming, adaptive immune evasion, or clonal evolution under therapeutic pressure, rather than pre-existing refractory states. Although primary and acquired resistance represent distinct clinical phenotypes, they are driven by overlapping biological mechanisms, including intrinsic resistance programs and adaptive immune evasion processes under therapeutic pressure (5, 32). These processes are driven by overlapping but context-dependent biological pathways and have different clinical implications for treatment sequencing. As summarized in **Figure 1**, resistance to PD-1/PD-L1 blockade manifests as two clinically distinct phenotypes, namely primary resistance and acquired resistance, which differ in onset timing, clinical trajectory, and underlying biological drivers. The following two subsections address primary and acquired resistance in turn.

**Figure 2:**
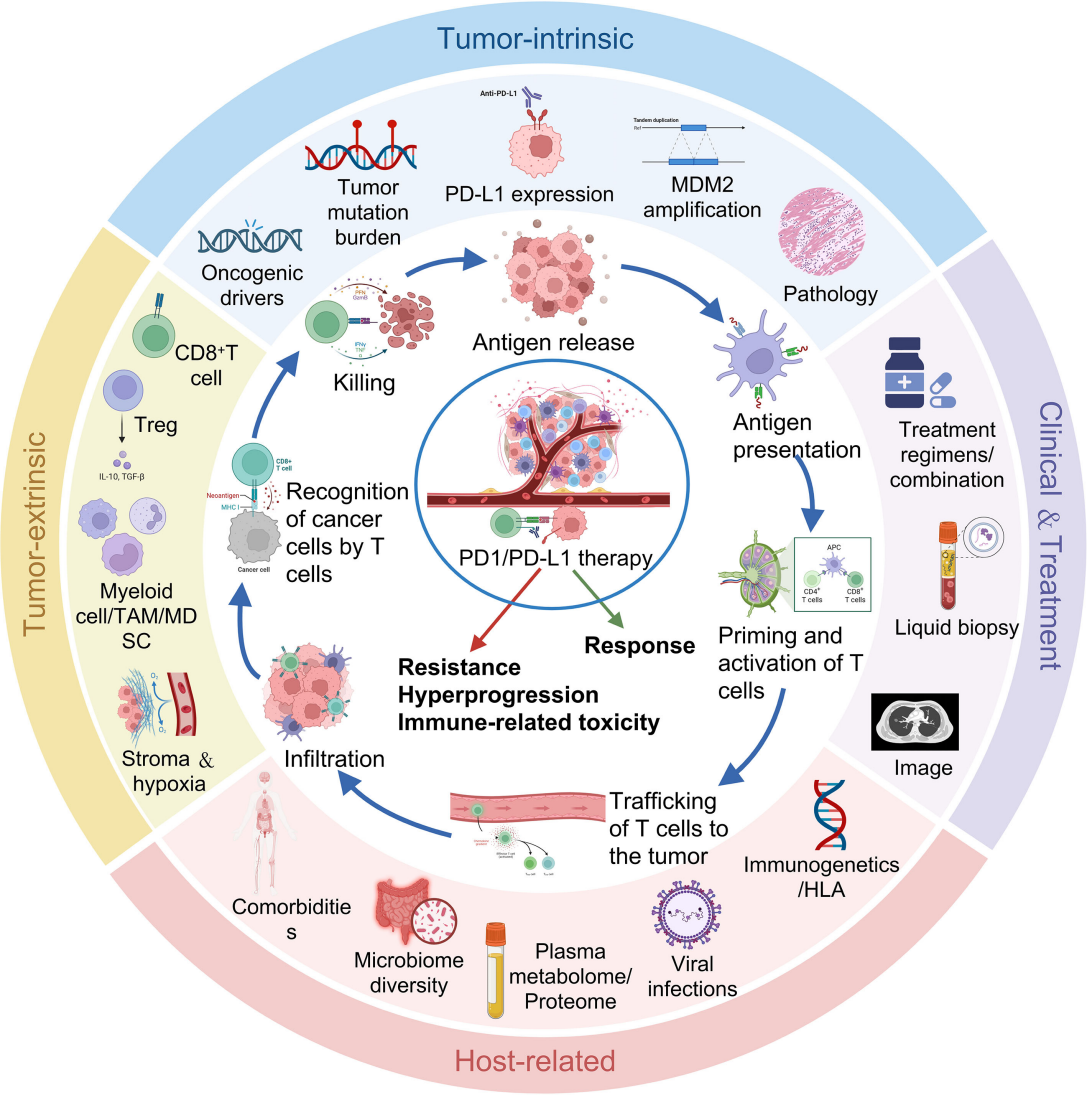
A unified biomarker system derived from determinants of resistance, HPD, and immune-related toxicity. Biomarkers are classified into four categories: tumor-intrinsic features, microenvironmental influences, clinical characteristics (including liquid biopsy parameters), and host-related factors. Although liquid biopsy parameters are grouped under clinical characteristics due to their sample source, their analytes may reflect alterations across the other three categories.

The biological determinants that drive these resistance phenotypes can be organized into a biomarker system based on four categories, as shown in **Figure 2**, namely tumor-intrinsic features, tumor-extrinsic microenvironmental influences, clinical characteristics, and host-related factors. The multi-omics landscape of resistance mechanisms underlying these categories is summarized in **Table 3**. Of note, clinical characteristics in this framework include routine medical history, performance status, laboratory tests, imaging features, and liquid biopsy parameters. While liquid biopsy parameters are grouped under clinical characteristics due to their blood-based sample source, their specific analytes may simultaneously reflect tumor-intrinsic alterations, microenvironmental remodeling, or host immune status. Importantly, this biomarker framework extends beyond resistance prediction, as these same categories of biomarkers are also associated with HPD and immune-related adverse events (irAEs), reflecting shared biological pathways across the three clinical phenomena.

**Table 3:** Multi-omics domains and mechanistic pathways underlying resistance to PD-1/PD-L1 inhibitors in lung cancer.

Omics layer	Representative biomarker patterns	Dominant biological mechanisms	Potential clinical implications
Genomics	Oncogene-addicted drivers with low tumor mutational burden; co-mutations in STK11/LKB1, KEAP1, and related tumor-suppressor genes; loss of β2-microglobulin or HLA class I molecules; rare baseline interferon-pathway defects (with JAK–STAT loss more canonically linked to acquired resistance)	Reduced neoantigen load; impaired interferon competence and antigen presentation; metabolic rewiring that favours immune evasion	Identification of patients unlikely to benefit from PD-1/PD-L1 monotherapy; prioritization of combination regimens with chemotherapy, radiotherapy, or targeted agents
Transcriptomics and immune gene signatures	Non-inflamed expression profiles with low interferon-γ signature, reduced chemokines recruiting effector T cells, and enrichment of myeloid or angiogenic programs	T-cell exclusion from the tumor core; expansion of immunosuppressive myeloid and stromal compartments; abnormal vasculature limiting lymphocyte trafficking	Refinement of PD-L1 and tumor mutational burden as predictors; rationale for adding anti-angiogenic or myeloid-directed therapies to PD-1/PD-L1 blockade
Epigenomics	Promoter methylation of antigen-processing and immune-regulatory genes; global CpG hypermethylation patterns; altered expression of chromatin-modifying enzymes	Transcriptional silencing of antigen-presentation machinery and T-cell chemoattractants; stable repression of interferon-responsive networks	Selection of patients for epigenetic priming strategies using DNA-methyltransferase or histone-deacetylase inhibitors prior to or concurrent with PD-1/PD-L1 therapy
Proteomics and phosphoproteomics	Low abundance of HLA class I and antigen-processing proteins; activation of NRF2, PI3K–AKT, and cell-cycle kinase pathways; increased expression of alternative immune checkpoints and ligands	Enhanced resistance to oxidative stress and cytotoxic attack; proliferative signalling that outpaces immune control; engagement of non–PD-1 inhibitory pathways	Support for rational kinase-inhibitor and checkpoint-inhibitor combinations; prioritization of targets for drug development within resistant molecular subgroups
Metabolomics and microbiome	Serum and tumor metabolite profiles dominated by lactate, adenosine, and altered amino-acid or lipid metabolism; dysbiotic gut or airway microbial communities with reduced diversity	Metabolic suppression of effector T cells; expansion of regulatory and myeloid-suppressor populations; systemic immune skewing away from effective antitumor responses	Identification of patients for metabolic or microbiome-modulating interventions, including inhibitors of adenosine or IDO pathways and microbiota-directed strategies
Integrated multi-omics models	Composite scores that combine genomic drivers, tumor mutational burden, immune and stromal transcriptomic signatures, epigenetic indices, proteomic surrogates, and circulating biomarkers	Systems-level representation of convergent tumor and host perturbations that encode primary resistance risk	Probabilistic prediction of primary resistance, stratification in immunotherapy trials, and design of adaptive treatment algorithms in routine practice

### 4.1 Primary resistance

Rather than analyzing genomics, transcriptomics, proteomics, and metabolomics as separate layers, current evidence suggests that primary resistance to PD-1/PD-L1 blockade is driven by multiple interacting factors present at baseline (5, 32). These factors include tumor-intrinsic and tumor-extrinsic determinants, which are the focus of this discussion. Clinical characteristics and host-related factors may also contribute but are not emphasized here.

At the tumor-intrinsic level, oncogene-driven tumors and specific co-mutation patterns are enriched among primary non-responders (71–76). In NSCLC, tumors harboring alterations in *EGFR*, *ALK*, or *KRAS* co-mutations such as *STK11*/*LKB1* or *KEAP1* are associated with low TMB, impaired antigen presentation, and reduced T-cell infiltration, thereby defining immune-cold tumor states that predict primary resistance (72–77). Loss-of-function mutations in beta-2-microglobulin (B2M) or *HLA* class I genes, although less common at baseline, also compromise antigen presentation and contribute to non-response (77). Transcriptomic and epigenetic profiling further differentiates inflamed versus non-inflamed immune states (76, 78). Interferon-γ–related gene signatures and chemokine programs correlate with response, whereas myeloid-dominant or TGF-β–driven programs and promoter methylation of antigen-processing genes are associated with immune pathway silencing and primary resistance (78, 79). Proteomic and phosphoproteomic analyses reveal activation of oncogenic signaling pathways (PI3K-AKT, RAS-MAPK), antioxidant programs such as NRF2, and reduced antigen presentation, all contributing to immune evasion (42).

At the tumor-extrinsic level, systemic factors, including circulating metabolites, plasma proteomics, and host immune-cell phenotypes, influence primary treatment outcomes (80). Serum metabolite profiles dominated by lactate, adenosine, and altered amino-acid or lipid metabolism have been linked to poor response to PD-1/PD-L1 blockade (81). These findings highlight the importance of host metabolic and inflammatory states, though this discussion focuses on their interaction with tumor-intrinsic and extrinsic mechanisms.

Composite scores integrating tumor-intrinsic and tumor-extrinsic factors, including genomic drivers, TMB, immune and stromal transcriptomic signatures, epigenetic indices, and circulating biomarkers, have shown improved performance over single-analyte predictors for stratifying patients by primary resistance risk (41). Most of these models, however, remain investigational and require prospective validation.

Methodologically, studies on primary resistance biomarkers are limited by retrospective designs, heterogeneous treatment settings, and insufficient external validation. Many reported models lack robust validation and calibration, raising concerns that their apparent predictive performance may reflect overfitting or cohort-specific biases rather than generalizable biology.

### 4.2 Acquired resistance

Acquired resistance develops gradually under the selective pressure of PD-1/PD-L1 blockade, in contrast to primary resistance, which is largely determined by pre-existing baseline features (5, 32). Despite distinct definitions, primary and acquired resistance involve largely overlapping molecular pathways, differing mainly in timing (5, 6, 29, 32, 75). Understanding these mechanisms requires longitudinal studies with serial multi-omics profiling, integrating genomic, transcriptomic, and immunophenotypic analyses.

At the tumor-intrinsic level, high-quality evidence indicates that acquired resistance is primarily driven by genetic alterations. In a paired pre- and post-treatment biopsy study of 82 patients, loss-of-function mutations in *JAK1*, *JAK2*, *STK11*, *B2M*, *APC*, *MTOR*, and *KEAP1* were identified in 27.8% of patients treated with ICIs. These alterations were absent in a control cohort receiving chemotherapy or targeted therapy (75). *JAK1/2* mutations directly disrupt IFN-γ receptor signaling, representing a core mechanism of IFN-γ pathway inactivation. This reduces MHC-I upregulation, PD-L1 expression, and T-cell responsiveness to interferon signals. Additional mutations, including *STK11*, *KEAP1*, *MTOR*, and *APC*, may indirectly impair IFN-γ signaling by altering tumor metabolism, redox balance, or antigen presentation (29). *B2M* truncating mutations or loss of heterozygosity further compromise antigen presentation, enabling escape from CD8^+^ T-cell recognition. These alterations are generally absent in pre-treatment biopsies, explaining their rarity in primary resistance and emergence after prolonged ICI therapy.

At the tumor-extrinsic level, post-progression tumors frequently show upregulation of alternative immune checkpoints, such as TIM-3, LAG-3, and VISTA (44, 82). These changes can limit T-cell effector function despite ongoing therapy (75, 83). Simultaneously, remodeling of the myeloid compartment occurs, including increased infiltration of M2-polarized tumor-associated macrophages (TAMs) and MDSCs. This establishes an immunosuppressive microenvironment that sustains tumor survival and growth (29, 83).

Emerging evidence suggests that epigenetic and metabolic adaptations may contribute to acquired resistance, although current data are limited and these mechanisms are mainly supported in the context of primary resistance. Alterations in DNA methylation at immune-related gene promoters, as well as shifts in T-cell metabolism toward fatty acid oxidation with reduced glycolysis, have been proposed to impair antitumor immunity following initial response. These mechanisms remain hypothetical or less well validated in NSCLC. While difficult to monitor directly, non-invasive approaches such as serial ctDNA profiling can provide complementary insights.

Liquid biopsy approaches integrated with multi-omics profiling provide a non-invasive means to track emerging resistance. Increases in ctDNA levels, emergence of *JAK2* or *B2M* mutations in plasma, or loss of previously detected response-associated mutations may indicate impending acquired resistance.

Methodologically, studies on acquired resistance biomarkers are limited by retrospective designs, small sample sizes, and lack of serial biospecimens. Many reported alterations have not been prospectively validated. The optimal timing and frequency of multi-omics and liquid biopsy sampling remain undefined. Given the dynamic nature of acquired resistance, on-treatment monitoring tools are essential for detection, tracking, and guiding therapeutic adaptation. Further discussion of these tools is provided in the following sections.

## 5 Multi-omics determinants of HPD during PD-1/PD-L1 therapy

HPD represents an extreme failure pattern of PD-1/PD-L1 blockade, characterized by a marked increase in tumor growth rate, early clinical deterioration, and substantially shortened survival compared with conventional progression (17, 18, 84). Although its definition varies across studies, it is typically assessed using tumor growth kinetics metrics such as tumor growth rate or tumor growth kinetics (17, 18). In advanced NSCLC, retrospective series using growth-kinetic metrics report HPD incidence between approximately 8% and 20% of patients treated with PD-1/PD-L1 inhibitors, with consistently inferior overall survival relative to non-HPD progressors (84). In a systems-immunology framework, HPD appears to arise from the convergence of aggressive clonal architecture, immune exclusion or myeloid dominance, metabolic reprogramming, and host inflammatory permissiveness rather than from a single genomic lesion (16). These observations indicate that HPD is not a random radiological phenomenon but a distinct biological state, and have prompted multi-omics efforts to decode tumor-intrinsic and microenvironmental determinants.

At the genomic level, copy-number alterations and oncogenic drivers emerge as recurrent features in HPD cohorts. Early pan-cancer sequencing studies linked amplification of *MDM2/MDM4* and *EGFR* aberrations to accelerated post-immunotherapy growth, and subsequent reviews in lung cancer have consolidated these lesions as candidate risk markers (85). Whole-exome sequencing of HPD versus non-HPD tumors under combined immunotherapy showed that canonical driver mutations were largely conserved between pre- and post-HPD samples (86). However, HPD cases displayed higher frequencies of *TP53* and *CNN2* mutations and significantly increased mutant-allele tumor heterogeneity. This suggests that pre-existing clonal complexity, rather than the acquisition of new drivers, predisposes to HPD. Case-based genomic analyses in NSCLC with *HER2* exon 20 insertion further illustrate this principle (87). Both reported HPD patients carried HER2 insertion plus broad co-amplifications in *FGF* and cell-cycle loci (*FGF3/FGF4/FGF19*, *CCND1*). They also had alterations in PI3K/AKT and cell-cycle signaling, together with low TMB and intermediate PD-L1 expression (85, 87, 88). These findings support a model in which specific oncogenic constellations and copy-number patterns generate an inherently aggressive, immune-evasive background in which PD-1/PD-L1 blockade can unmask rapid outgrowth.

Transcriptomic and immunogenetic profiling refine these genomic observations by delineating pathway-level dysregulation and immune-microenvironment context in HPD. In a multi-omics study of paired tumors from patients receiving combination immunotherapy, integration of RNA sequencing with multiplex immunofluorescence revealed upregulation of IL6 mRNA in post-HPD samples (89). It also showed a more immunosuppressive tumor microenvironment with increased M2/M1 macrophage ratio and higher mutant-allele heterogeneity relative to non-HPD controls. *In vitro* models derived from baseline and HPD biopsies of NSCLC patients treated with PD-1/PD-L1 inhibitors provide additional insights (90). HPD-associated cell lines acquire enhanced proliferative capacity, develop epithelial-mesenchymal transition and stemness features, maintain persistent PD-L1 and CD44 expression, and show hyperactivation of ERK/MAPK and IFN-γ pathway components such as IFNGR1. These studies indicate that chronic IFN-γ/PD-L1 signaling and transcriptional plasticity within tumor cells can drive a shift toward more aggressive, therapy-refractory states rather than simply restoring antitumor immunity. Complementary systems-level work has integrated transcriptomic, metabolic, and immune profiling across tumor models (91). This work suggests that changes in tumor metabolism and inflammation driven by myeloid cells, particularly through the IL-6/STAT3 pathway and related signaling, play a central role in linking immune checkpoint blockade to hyperaccelerated tumor growth.

Beyond tissue-based omics, non-invasive multi-omics approaches are beginning to address the need for early HPD prediction and discrimination from pseudoprogression. Radiomic analyses of baseline CT scans in advanced NSCLC have identified texture- and vasculature-based features that classify patients at risk of HPD with high discriminatory performance, and subsequent multi-center work has confirmed that radiomic signatures combined with clinical variables can distinguish HPD, standard progression, and atypical response patterns (92, 93). Recent reviews integrating genomics, transcriptomics, proteomics, microbiomics and radiomics in NSCLC immunotherapy propose that HPD likely occupies a specific region within a broader multi-dimensional response space, characterized by unfavorable oncogenic and copy-number configurations, non-inflamed or myeloid-dominant transcriptional programs, systemic inflammatory mediators such as IL-6, and imaging features of highly proliferative, heterogeneous tumors (16, 94). These observations collectively support the development of composite multi-omics risk models that explicitly include HPD as a distinct endpoint and that integrate longitudinal genomic, transcriptomic, immune-cell, soluble, and radiomic measurements to enable pre-treatment risk stratification and very early on-treatment identification of patients in whom PD-1/PD-L1 blockade is likely to trigger catastrophic HPD.

Methodologically, HPD biomarker studies are especially vulnerable to bias. Event counts are small, definitions vary across studies, and pretreatment growth trajectories are often unavailable, making it difficult to distinguish a true treatment-associated HPD state from intrinsically aggressive disease (17, 18, 94). Tissue obtained after explosive progression may reflect end-stage biology rather than causality, while radiomic signatures are sensitive to scanner heterogeneity and segmentation choices. In addition, publication bias toward positive findings may further inflate reported associations, while the lack of prospective validation limits the generalizability and clinical applicability of these biomarkers. Accordingly, current genomic, transcriptomic, and radiomic HPD markers should be regarded as hypothesis-generating until harmonized endpoints, prospective sampling windows, and independent external validation are achieved (16, 64, 66, 70, 94).

## 6 Multi-omics signatures of immune-related toxicity in lung cancer immunotherapy

Immune-related adverse events during PD-1/PD-L1 blockade in lung cancer are thought to reflect dysregulated host immune responses in the context of tumor–immune interactions that are only partially captured by routine clinical or laboratory markers (65). From a systems-biology perspective, irAE susceptibility can also be organized along four interacting axes: baseline immune activation, antigen-presentation genetics, tissue-resident effector programs, and systemic metabolic or microbial conditioning. Multi-omics studies across genomics, transcriptomics, proteomics, metabolomics and the microbiome indicate that irAEs correspond to a composite immunological state that overlaps with, but is distinct from, the signatures of antitumor efficacy, favoring composite predictors rather than single biomarkers (95).

A pan-cancer multi-omics analysis integrating pharmacovigilance data with The Cancer Genome Atlas demonstrated that tumor-intrinsic immune activation features, including CD8^+^ T-cell abundance, T-cell receptor diversity, cytolytic activity and interferon-γ–related gene-expression signatures, correlate positively with the reporting odds of irAEs under anti-PD-1/PD-L1 therapy (96). In the same study, expression of lymphocyte cytosolic protein 1 (LCP1) and adenosine diphosphate–dependent glucokinase (ADPGK) formed the best-performing bivariate predictor of irAE risk, with validation in a cohort dominated by lung cancer and particular utility for pneumonitis prediction. These findings suggest that highly inflamed, T cell–activated tumors are primed for both durable response and systemic auto-inflammatory toxicity.

Host germline genetics further modulate this propensity. In NSCLC treated with single-agent PD-1/PD-L1 inhibitors, high-resolution HLA typing showed that homozygosity at one or more class I loci was associated with a lower probability of pneumonitis or other grade ≥3 irAEs, but also with diminished survival benefit, whereas the HLA-A*03 supertype conferred increased irAE risk (97). A complementary analysis in high-PD-L1 NSCLC linked specific germline HLA class I and II haplotypes to checkpoint inhibitor pneumonitis and treatment response, reinforcing the view that antigen-presentation breadth influences both on-target tumor immunity and off-tumor tissue injury (98).

Tissue- and cell-level omics provide mechanistic resolution, particularly for lung toxicity. Single-cell RNA sequencing of bronchoalveolar lavage fluid from patients with checkpoint inhibitor–related pneumonitis reveals enrichment of activated CD8^+^ effector and tissue-resident cytotoxic T cells (99). In parallel, CD4^+^ T-helper populations display Th17-skewed transcriptional programs with upregulated migration and metabolic pathways. These features are clearly distinct from those observed in infectious or COVID-19 pneumonia. Similar single-cell analyses of diverse irAEs have identified shared phenotypes of clonally expanded, polyfunctional effector T cells and pro-inflammatory monocyte or macrophage subsets (100). As shown in **Table 4**, these tissue-resolved signatures complement germline and bulk-tumor biomarkers by linking defined immune-cell states and trafficking programs to organ-selective toxicity patterns.

**Table 4:** Multi-omics layers and candidate biomarker signatures associated with immune-related adverse events during PD-1/PD-L1 therapy in lung cancer.

Omics layer	Representative biomarker patterns	Dominant biological or clinical signals	Potential clinical implications
Germline and host genetics	HLA class I zygosity; specific HLA supertypes and haplotypes; selected immune-related polymorphisms	Variation in antigen-presentation breadth and self-peptide repertoire; differential propensity to develop pneumonitis or high-grade systemic irAEs	Pre-treatment identification of patients at low or high risk of severe toxicity; potential integration with efficacy predictors to inform treatment intensity
Tumor genomics and transcriptomics	High CD8^+^ T-cell infiltration; increased TCR diversity; interferon-γ and cytolytic gene-expression signatures; elevated LCP1 and ADPGK expression	Strong baseline T-cell activation and immune-inflamed microenvironment; heightened capacity for on-target tumor killing coupled to systemic immune activation	Refinement of PD-L1 and tumor mutational burden as toxicity and efficacy markers; selection of patients for intensified monitoring or prophylactic immunomodulation
Single-cell and spatial omics in lung tissue	Expanded effector and tissue-resident CD8^+^ T cells; Th17-skewed CD4^+^ subsets; activated monocyte and macrophage populations; altered local metabolic pathways	Local feed-forward inflammatory circuits and cell–cell interactions that drive checkpoint inhibitor–related pneumonitis	Mechanistic stratification of pneumonitis endotypes; rational prioritization of targeted immunosuppression (for example IL-17/IL-23 or GM-CSF axis blockade)
Plasma proteomics, metabolomics and lipidomics	Panels of inflammatory, complement and acute-phase proteins; amino-acid and energy-metabolism metabolites; specific phospholipid and neutral-lipid species	Systemic immune activation, metabolic stress and lipid-remodelling states that predispose to irAEs or reflect subclinical toxicity	Minimally invasive risk scores for baseline stratification and early on-treatment detection of emerging irAEs; potential guidance of dose modification and corticosteroid-sparing strategies
Microbiome and microbial metabolomics	Gut microbial diversity; enrichment or depletion of defined commensal taxa; microbial pathways producing short-chain fatty acids, bile acids and tryptophan metabolites	Modulation of systemic immune tone and mucosal barrier integrity; differential risk of gastrointestinal and systemic irAEs	Opportunities for microbiota-targeted interventions (diet, probiotics, faecal microbiota transplantation) to mitigate toxicity while maintaining antitumor immunity
Integrated multi-omics models	Composite scores combining germline markers, tumor immune-infiltration metrics, circulating proteins and metabolites, and microbiome features	Systems-level representation of host–tumor–microbiome interactions that encode both efficacy and toxicity risk	Development of clinically deployable algorithms for personalised PD-1/PD-L1 therapy selection, dynamic risk reassessment and rational design of de-escalation or escalation strategies

Circulating multi-omics biomarkers are particularly attractive for both baseline risk estimation and longitudinal monitoring in lung cancer. In older patients with advanced NSCLC, baseline plasma proteomic and metabolomic profiling identified panels of inflammatory proteins and small-molecule metabolites that stratified the risk of high-grade irAEs during PD-1/PD-L1 therapy and chemo-immunotherapy while also associating with survival (101). Independent lipidomic profiling in NSCLC revealed that a defined set of phospholipids and neutral lipids measured before treatment could predict subsequent irAEs with clinically relevant discrimination, implicating systemic lipid metabolism in conditioning toxicity risk (102). Multi-omics studies of the gut microbiome using metagenomics and metabolomics have linked enrichment of specific commensal taxa and microbial metabolic pathways to heightened risk of immune-mediated colitis or protection from severe toxicity (103), although some NSCLC-focused cohorts have not observed robust microbiome-toxicity associations, indicating disease- and regimen-specific effects.

These studies support a model in which immune-related toxicity during PD-1/PD-L1 therapy in lung cancer emerges from the intersection of pro-inflammatory tumor immune contexture, host antigen-presentation genetics, tissue-resident effector T-cell programs and systemic metabolic and microbial states. Multi-omics signatures integrating these layers offer a rational basis for pre-treatment risk stratification, organ-specific toxicity prediction and dynamic monitoring of subclinical immune activation.

Methodologically, irAE studies are limited by organ-specific endpoint heterogeneity, variable grading practices, and confounding from corticosteroids, antibiotics, intercurrent infection, and regimen selection. In addition, the lack of baseline autoantibody data in many studies limits the ability to account for pre-existing autoimmune predisposition. High-grade events are relatively infrequent, which encourages unstable feature selection when dozens to thousands of analytes are screened. Future multi-omics toxicity models should therefore report organ-specific calibration, competing-risk considerations, and prospective validation rather than only pooled discrimination metrics (65, 104).

## 7 Translational integration of multi-omics biomarkers: risk stratification, patient selection, dynamic monitoring, and trial design for PD-1/PD-L1 therapy

Translating multi-omics biomarkers into clinical practice requires a stepwise framework that spans baseline risk stratification, on-treatment dynamic monitoring, and post-progression decision-making. As a challenging field, we must acknowledge the gap between current knowledge and true translational implementation. Within these limitations, we pay particular attention to multi-omics findings closest to clinical actionability and, based on the growing body of evidence, propose clearer decision nodes for when and how to act on emerging biomarker signals (64, 66, 70). In fact, despite the overall thin evidence base, resistance, particularly acquired resistance, offers the strongest clinical actionability, best exemplifies the value of dynamic monitoring, and represents the area where current translational efforts are most concentrated (6, 29, 32, 75). We therefore proceed to discuss baseline risk stratification (Section 7.1), efficacy-risk trade-off (Section 7.2), dynamic monitoring and acquired resistance (Section 7.3), clinical case illustration (Section 7.4), and trial design implications (Section 7.5).

### 7.1 Baseline risk stratification

Translating multi-omics biomarkers into clinical decision tools requires explicit linkage between molecular signatures and concrete therapeutic choices for PD-1/PD-L1 therapy in lung cancer. Composite models that integrate tumor genomics, transcriptomics, epigenomics, proteomics, metabolomics, radiomics, and microbiome features with clinical variables and performance status may improve the estimation of treatment benefit, HPD risk, and immune-related toxicity beyond single-analyte markers such as PD-L1 or TMB, pending validation (105). However, because efficacy-related outcomes and treatment-related toxicity are governed by partially overlapping but distinct biological processes, multi-omics signatures are likely to be most useful when integrated into separate risk-stratification models for efficacy and safety. In this framework, one set of models may be developed to predict tumor-dominant failure states such as primary or acquired resistance and HPD, whereas another may be optimized to predict toxicity-related outcomes such as irAEs. The supporting evidence remains uneven across these domains, and simultaneous prediction of resistance, HPD, and irAEs within a single unified model remains difficult with current data and methodologies. **Table 5** summarizes the clinical translation of multi-omics biomarkers for PD-1/PD-L1 therapy in lung cancer, covering baseline risk stratification, patient selection, dynamic monitoring, and trial design.

**Table 5:** Clinical translation of multi-omics biomarkers for PD-1/PD-L1 therapy in lung cancer.

Translational objective	Multi-omics biomarker inputs	Example implementation in PD-1/PD-L1 therapy	Anticipated clinical impact
Baseline risk stratification for primary resistance	Tumor genomics (drivers, co-mutations), mutational burden, immune and stromal transcriptomic signatures, epigenetic indices, proteomic surrogates of antigen presentation	Pre-treatment composite score reported with pathology to classify patients into high-, intermediate-, and low-benefit groups before initiating PD-1/PD-L1 inhibitors	More selective use of monotherapy, prioritization of chemo-immunotherapy or alternative regimens in high-resistance strata
Identification of HPD-prone subgroups	Copy-number patterns, oncogenic constellations, myeloid- and IL-6–related transcriptional programs, baseline radiomic growth-kinetic features	Pre-specified “HPD-risk” module incorporated into multi-omics report and used as a stratification factor in PD-1/PD-L1 trials	Early diversion of high-risk patients to non–PD-1/PD-L1 strategies or intensified early imaging surveillance
Prediction of immune-related toxicity	Germline HLA metrics, tumor-intrinsic immune-activation signatures, single-cell or bulk immune-cell states, plasma proteomic and metabolomic panels, microbiome profiles	Baseline toxicity-risk index guiding regimen choice, monitoring intensity, and thresholds for corticosteroid initiation	Reduction of high-grade irAEs and unplanned treatment discontinuation while preserving efficacy in tolerable-risk groups
Dynamic response monitoring	Serial ctDNA, blood-based transcriptomic and proteomic signatures, metabolomic changes, longitudinal radiomics	On-treatment multi-omics re-assessment at early timepoints to reclassify patients as molecular responders, non-responders, or putative HPD	Timely switching from ineffective PD-1/PD-L1 therapy, minimization of exposure in molecular non-responders, and improved identification of durable responders
Trial design and endpoint definition	Integrated models combining baseline and longitudinal multi-omics features with survival outcomes	Use of multi-omics scores for biomarker-enriched inclusion, stratified randomization, and co-primary endpoints in PD-1/PD-L1 trials	Increased statistical efficiency, clearer signal detection in biologically relevant subgroups, and more rapid validation of composite biomarkers

**Figure 3:**
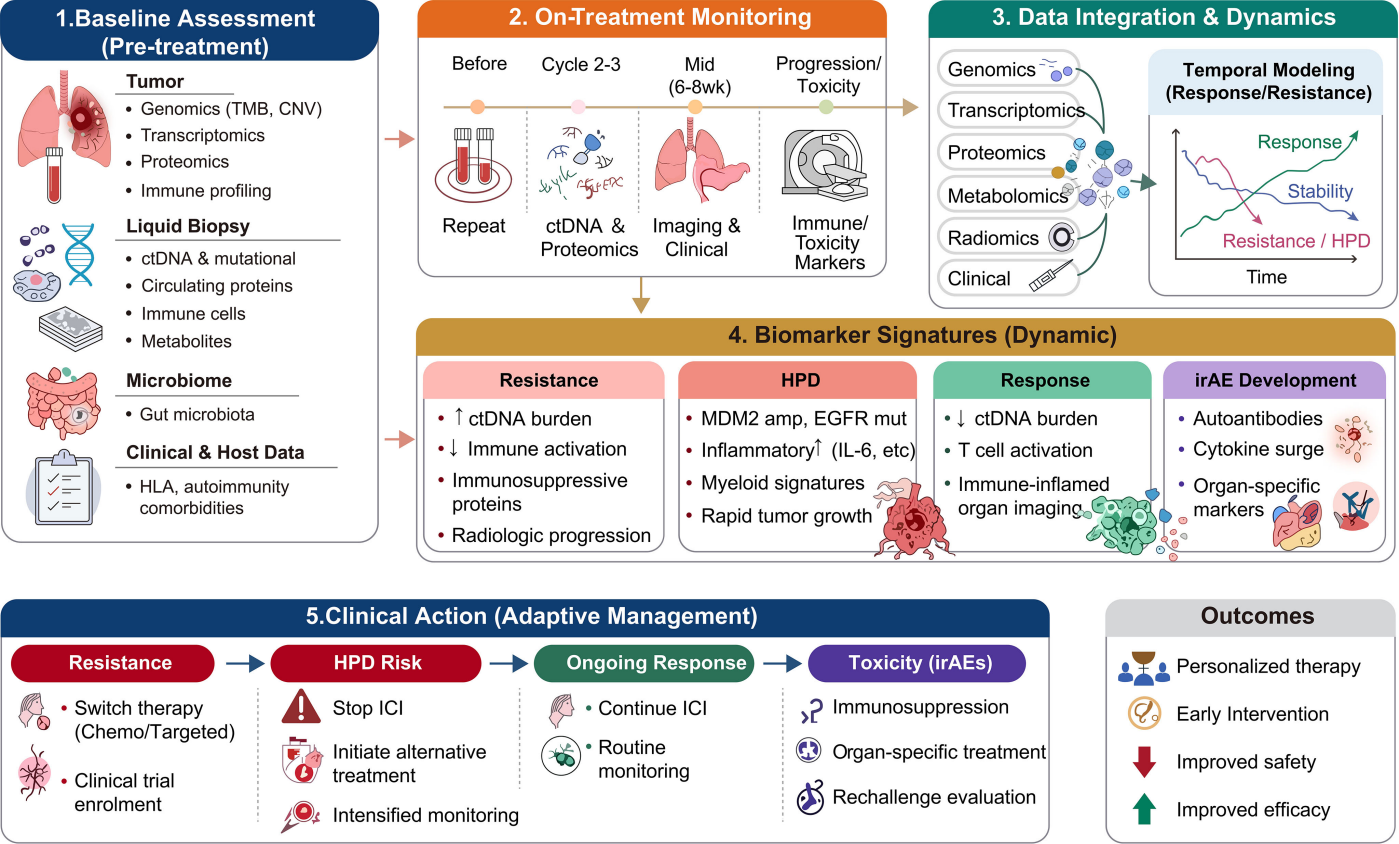
Multi-omics integration framework for predicting immunotherapy outcomes and patient stratification. The framework integrates baseline assessment (tumor, liquid biopsy, microbiome, clinical/host data), on-treatment dynamic monitoring (serial ctDNA, imaging, proteomics), temporal data integration, and dynamic biomarker signatures to guide adaptive clinical management. Based on the identified state (resistance, HPD, response, or toxicity), corresponding actions include therapy switch, ICI discontinuation, continued treatment, or immunosuppression with rechallenge evaluation. The framework ultimately supports personalized therapy, early intervention, improved safety, and improved efficacy.

In the pre-treatment setting, multi-omics predictors derived from bulk and single-cell transcriptomics, copy-number and mutation profiles, methylation patterns, immune deconvolution, and circulating proteins have already been used to construct prognostic and immunotherapy-response scores in NSCLC, including signatures that enrich for patients with inflamed microenvironments, programmed cell death pathway activation, or adverse metabolic-stromal programs (49, 106). Baseline plasma proteome-based classifiers illustrate that liquid-biopsy multi-omics can support first-line treatment selection when tissue is limited or when repeated invasive sampling is impractical (49, 107). **Figure 3** presents a multi-omics integration framework for predicting immunotherapy outcomes and patient stratification. The framework integrates baseline multi-dimensional assessment, on-treatment dynamic monitoring, temporal data integration, and dynamic biomarker signatures to guide adaptive clinical management. Based on the identified states (resistance, HPD, response, or toxicity), corresponding clinical actions include therapy switch, ICI discontinuation, continued treatment with routine monitoring, or immunosuppression with rechallenge evaluation. Such scores can be operationalized to allocate patients into biomarker-defined strata: those suitable for PD-1/PD-L1 monotherapy, those requiring intensified combination regimens because of high primary resistance probability, and those in whom elevated risk of HPD or severe irAEs may favor alternative strategies or modified dosing. In practice, this implies embedding validated composite scores into electronic decision-support systems and reporting them alongside PD-L1 and standard clinical staging.

### 7.2 Navigating the efficacy–risk trade-off

A more nuanced challenge arises when efficacy and risk predictions point in opposite directions, a scenario where the same patient is classified as both high probability for durable response and high risk for severe adverse outcomes such as severe irAEs or HPD (10, 11, 96). This “high-efficacy/high-risk” dilemma is not uncommon, given that inflamed tumor immune microenvironments predispose to both antitumor response and autoimmune toxicity. Current evidence does not support a simple formula to resolve this trade off, but multi-omics can reframe the decision from a binary “treat or not” to a stratified risk management strategy (108). For the high-efficacy/high-risk group, we propose three steps: (i) joint probability reporting that explicitly categorizes patients into four efficacy-risk quadrants; (ii) mitigation strategies guided by multi-omics, including intensified monitoring, modified dosing, or investigationally prophylactic immunomodulation; and (iii) dynamic reassessment to detect early molecular response or subclinical toxicity, enabling preemptive intervention. Ultimately, the field should move toward a joint benefit–risk composite score that weights efficacy and toxicity probabilities by clinical severity, requiring prospective trials that capture patient-centered outcomes alongside multi-omics measurements.

### 7.3 Dynamic monitoring and technical challenges: from on-treatment tracking to acquired resistance detection

Among the three clinical outcomes of immunotherapy, namely resistance, HPD, and irAEs, acquired resistance offers the strongest near-term clinical actionability (29). This is attributable to the largest body of research evidence and the methodological advantage of paired biopsy designs, as well as the pressing clinical need that over 60% of initial responders eventually develop acquired resistance, and that post-progression therapeutic decisions themselves remain challenging and controversial. This need is particularly salient given that first-line regimens have become largely standardized (2). In contrast, HPD lacks validated biomarkers, and irAEs require a fundamentally different management paradigm. We therefore focus on acquired resistance in the following discussion.

Dynamic monitoring represents a second pillar of translational integration. Serial liquid-biopsy-based multi-omics approaches that couple ctDNA kinetics with multiplexed profiling of circulating immune cells, soluble factors, and metabolites have been shown to correlate with radiographic response and survival under anti-PD-1 therapy in metastatic NSCLC, and to yield blood “immunomaps” that distinguish durable responders from early progressors (62, 63, 109). Beyond mutation-based ctDNA tracking, cfDNA methylation profiling offers a complementary tissue-agnostic approach; a decrease in ctDNA methylation score from baseline to cycle 3 of pembrolizumab has been associated with higher response rates and longer survival across multiple solid tumors, and in NSCLC, early methylation changes can predict response ahead of radiographic imaging (110, 111). These frameworks are relevant not only to early pharmacodynamic response assessment but also to discrimination between primary resistance, emerging acquired resistance, incipient HPD, and evolving toxicity. However, a critical challenge remains: distinguishing true molecular progression from transient ctDNA elevation caused by therapy-induced tumor inflammation or necrosis, which can mimic pseudoprogression at the circulating tumor DNA level (47, 112). Misinterpretation may lead to premature treatment discontinuation in responding patients or delayed switching in truly progressive disease. Several strategies can help address this challenge. First, applying quantitative thresholds rather than qualitative “any increase” criteria, such as requiring a predefined fold rise (e.g., ≥2 fold) or an absolute increase in variant allele frequency, improves specificity. Second, integrating ctDNA dynamics with radiographic features, particularly metabolic changes on ¹^8^F-FDG PET, can help differentiate inflammatory flares from true progression. Third, complementary circulating biomarkers, including cytokines (e.g., IL-8) or circulating tumor cell phenotyping, may provide additional discriminatory information. Prospective studies are needed to validate these integrated approaches before routine clinical adoption.

Reliable detection of acquired resistance mechanisms depends heavily on post-progression tissue or liquid re-biopsy (2, 47). Tissue re-biopsy remains the gold standard, but liquid biopsy offers a minimally invasive alternative that can capture multi-lesion heterogeneity (47). For acquired resistance detected at progression, multi-omics signals can be ranked into three tiers based on evidence maturity and clinical actionability. Tier 1 includes acquired loss-of-function mutations in *B2M* or *JAK1/2* detected on post-progression re-biopsy (72, 75); these alterations predict failure of PD-1/PD-L1 rechallenge and support discontinuation of immunotherapy with switch to chemotherapy or clinical trials. Tier 2 includes upregulation of alternative immune checkpoints such as LAG3, TIGIT, or TIM3 (44), which have shown promise in clinical trials but mainly in melanoma rather than lung cancer, and cfDNA methylation dynamics that correlate with early response but lack validated thresholds for clinical action (110, 111). Tier 3 comprises emerging signals requiring prospective validation, including epigenetic reprogramming signatures such as EZH2-mediated H3K27me3 (78) and plasma proteomic or AI-based transcriptomic models (64, 66, 101). This ranking reflects a hierarchy of validation: Tier 1 signals have multi-cohort validation and clear action paths; Tier 2 signals are clinically established but with suboptimal performance or await lung-specific validation; Tier 3 signals require further prospective validation, highlighting the need for continued multi-omics research.

Based on this framework, we propose a pragmatic clinical workflow. The primary trigger for treatment change should remain radiographic progression per RECIST 1.1; upon confirmed progression, post-progression tissue biopsy and simultaneous plasma collection for ctDNA-based multi-omics should be obtained, with liquid biopsy serving as an alternative when tissue is unavailable. Once results are available, a Tier 1 signal (e.g., *B2M* or *JAK1/2* mutations) should lead to discontinuation of current anti-PD-1/PD-L1 therapy and referral to a mechanism-matched clinical trial or switch to standard chemotherapy, whereas if only Tier 2 or Tier 3 signals are detected, standard chemotherapy is recommended with consideration of histologic transformation (2). For patients without radiographic progression but with positive liquid biopsy signals (e.g., rising ctDNA or emergence of resistance-associated mutations), current evidence is insufficient to recommend a change in systemic therapy; however, such signals should trigger repeat liquid biopsy in 2–4 weeks, shortened imaging intervals to 4–6 weeks, and integration with clinical assessment (symptoms, LDH trends) to detect early progression and avoid delayed intervention. Consideration of prospective clinical trials evaluating resistance-guided preemptive interventions is encouraged. For oligoprogression (e.g., 1–5 progressive lesions) with a resistance signal, local ablative therapy combined with continued immunotherapy is a viable strategy (2, 27).

Several major gaps remain. First, no prospective trial has shown that acting on post-progression multi-omics improves outcomes; dedicated pragmatic trials are urgently needed. Second, there is no consensus on what constitutes a “positive” resistance signature; multi-center consortia should establish standardized guidelines. Third, only a minority of progressing patients undergo re-biopsy; streamlining re-biopsy pathways and promoting liquid biopsy are necessary. Fourth, most clinical laboratories do not offer panels covering *B2M*, *JAK1/2*, or methylation markers; low-cost targeted panels are a priority. Fifth, no drugs are approved for acquired resistance to PD-1/PD-L1 therapy in lung cancer, underscoring the need for accelerated drug development. Addressing these gaps will require coordinated efforts; until then, the proposed framework should be viewed as a hypothesis-generating roadmap.

### 7.4 Clinical case illustration

Although artificial intelligence-driven multi-omics models and clinical decision support systems remain investigational and real-world application still relies heavily on clinician judgment, a clinical case illustrates how current multi-omics principles can already integrate into a unified workflow. Consider a 65-year-old male with advanced lung adenocarcinoma (PD−L1 60%, TMB−H, STK11 wild type) whose baseline liquid biopsy unexpectedly detected MDM2 amplification. Conventional PD−L1 and TMB would favor pembrolizumab monotherapy, but the MDM2 alteration signals elevated HPD risk. Applying the efficacy–risk quadrant framework, this patient falls into the “high efficacy/high risk” category, with favorable efficacy predictors yet elevated risk of a specific treatment failure mode, hyperprogressive disease. The integrated multi−omics profile therefore guided three decisions: (i) avoidance of standard dose monotherapy; (ii) selection of pembrolizumab–chemotherapy combination to mitigate HPD risk; and (iii) early ctDNA monitoring at week 3. A sharp decline in MDM2 and KRAS circulating tumor DNA fractions confirmed molecular response, supporting continued treatment. This case demonstrates how multi−omics can link baseline risk stratification, regimen selection, and dynamic monitoring into a coherent clinical action plan.

### 7.5 Implications for clinical trial design

For clinical trials, multi-omics biomarkers enable rational enrichment, stratification, and adaptive design. Lung cancer biomarker trials already use genomic and PD-L1-based enrichment; extension to multi-omics signatures allows enrollment of patients who are molecularly poised to benefit from PD-1/PD-L1 therapy, or conversely, those at high risk of primary resistance for trials testing intensified combinations (61, 105). Biomarker-adaptive and group-sequential enrichment designs can exploit interim analyses of multi-omics-defined subpopulations to expand or restrict accrual while preserving statistical validity, thereby accelerating validation of composite biomarkers and reducing exposure of unlikely responders (113). Multi-omics scores may also serve as co-primary or hierarchical secondary endpoints, particularly when capturing long-term survival or durable clinical benefit.

## 8 Translational readiness and evidence levels

At present, the clinically implemented segment of this ecosystem is dominated by analytically validated genomic or immunohistochemical assays rather than fully integrated multi-omics platforms. PD-L1 immunohistochemistry and commercial comprehensive genomic profiling assays, including FoundationOne CDx, FoundationOne Liquid CDx, Guardant360 CDx, NeoGenomics PanTracer LBx, and GeneseeqPrime, provide standardized testing for established therapeutic biomarkers and selected complex features; however, they do not yet deliver integrated probabilities of primary resistance, HPD, or irAEs (109, 114–117). Publication-based multi-omics tools, including blood immunomaps, radiogenomic classifiers, and plasma proteome algorithms, cover broader biology, yet most remain investigational, platform-specific, or laboratory-developed models awaiting locked external validation (49, 63, 107).

Translational readiness therefore depends on more than biological plausibility, encompassing assay robustness, reproducibility, and cost considerations. Assays must demonstrate pre-analytic stability, inter-laboratory reproducibility, cross-platform concordance, scalable turnaround times, acceptable per-patient cost, and regulatory-grade software governance when algorithmic components are used. Small retrospective cohorts with thousands of candidate features are especially prone to overfitting, optimistic internal validation, and hidden confounding by regimen selection, steroid use, antibiotic exposure, or tumor burden. For this reason, biomarkers supported only by retrospective discovery studies should be distinguished from those with multi-cohort replication, prospective validation, or clinical implementation (64–66, 70). An evidence-level summary is provided in **Table 6**.

**Table 6:** Evidence-level and translational-readiness framework for multi-omics biomarker classes discussed in this review.

Application	Representative biomarker classes	Evidence-level category*	Current readiness	Main barriers before routine use
Baseline primary-resistance stratification	Integrated tissue genomics, transcriptomics, epigenomics, and immune-exclusion signatures	Retrospective multi-cohort validation	Investigational decision support / trial enrichment	Cross-platform harmonization; regimen heterogeneity; locked prospective validation
Baseline circulating multi-omics	Plasma proteome, transcriptome, metabolome, and blood immune-cell panels	Retrospective multi-cohort validation	Emerging analytical validation; limited deployment	Pre-analytic handling; inter-laboratory reproducibility; reimbursement and regulatory pathway
HPD prediction	Copy-number and driver constellations, myeloid or IL-6 programs, radiomic growth-kinetic models	Retrospective discovery cohort	Exploratory only	Rare event rates; non-uniform HPD definitions; limited paired pre/post-treatment sampling
irAE prediction	HLA or germline markers, plasma proteomic or metabolomic panels, microbiome features	Retrospective discovery cohort	Exploratory organ-specific risk modeling	Organ-specific endpoint heterogeneity; steroid and infection confounding; few high-grade events
Dynamic monitoring	Serial ctDNA kinetics with circulating immune, protein, metabolite, and radiomic features	Retrospective multi-cohort validation	Early clinical-trial use, not routine care	Sampling cadence; tumor shedding variability; integration with imaging and action thresholds
Currently implemented comparators	PD-L1 IHC and validated tissue or cfDNA comprehensive genomic profiling assays	Clinically implemented	Routine for selected treatment decisions, but not full multi-omics	Limited coverage of HPD and irAE biology; no unified cross-modality risk estimate

Implementing these strategies in routine practice therefore requires standardized pre-analytic workflows, cross-platform calibration, and rigorous analytical and clinical validation of multi-omics tests, ideally using prospective PD-1/PD-L1 lung cancer cohorts with pre-specified hypotheses. High-resolution multi-omics and single-cell technologies must be coupled to scalable machine-learning pipelines that remain interpretable to clinicians and regulators, and future studies should report calibration, action thresholds, missing-data handling, and health-economic impact alongside discrimination metrics (64–66, 70). Translational integration will depend on demonstrating that multi-omics-guided risk stratification, dynamic monitoring, and biomarker-driven trial designs improve patient-centered outcomes and cost-effectiveness compared with current PD-L1- and clinicopathologic-based algorithms. Crucially, future machine learning models must prioritize interpretability, enabling clinicians to understand which features drive a prediction, and demonstrate robust generalizability across diverse populations, sequencing platforms, and treatment settings before clinical deployment.

## 9 Future perspectives and concluding remarks

Future work on multi-omics biomarkers for PD-1/PD-L1 therapy in lung cancer will need to move from retrospective, single-center analyses toward large, prospective cohorts with harmonized definitions of primary resistance, HPD, and immune-related adverse events. Integrated genomic, transcriptomic, proteomic, metabolomic, radiomic, and microbiome datasets linked to standardized response and toxicity endpoints in NSCLC and SCLC are required to build and validate composite risk models that can be generalized across platforms and treatment regimens. Multi-omics and machine-learning frameworks in solid tumors already show that combined signatures outperform traditional single biomarkers for predicting long-term survival and durable benefit after immune checkpoint blockade.

Methodological priorities include robust feature selection, explicit control of overfitting, mitigation of small-cohort bias, transparent reporting of model calibration and clinical utility, and rigorous external validation across multi-ethnic populations. Work in NSCLC and pan-cancer cohorts demonstrates that multi-omics indicators integrating tumor-intrinsic alterations, immune activation markers, and systemic inflammatory or metabolic profiles can stratify patients by probability of durable benefit and early progression under PD-1/PD-L1 blockade, but external validation across independent lung cancer datasets remains limited (21, 50). Dedicated analyses should explicitly incorporate HPD as an outcome, combining genomics, immunogenetics, and radiomics to refine prediction of rapid progression phenotypes beyond conventional progression-free survival, and should examine whether such models add value over existing clinical and imaging-based predictors in NSCLC.

For immune-related toxicity, multi-omics strategies that integrate tumor immune activation signatures, germline or HLA-associated risk, plasma proteomic and metabolomic panels, and microbiome features have already yielded promising predictors of global and organ-specific irAEs under PD-1/PD-L1 therapy. Future studies in lung cancer should test whether combined efficacy–toxicity scores can support truly personalized benefit–risk estimation, for example by identifying patients who are simultaneously unlikely to benefit and at high risk of HPD or severe pneumonitis, and by informing tailored monitoring or prophylactic immunomodulation strategies. Implementation will depend on translating complex signatures into analytically validated assays, defining clinically interpretable thresholds, and demonstrating that multi-omics-guided decisions improve survival, toxicity, and resource utilization relative to PD-L1 and clinicopathologic algorithms alone.

## 10 Limitations

Several limitations constrain the current evidence base reviewed here. Definitions of resistance and especially HPD remain heterogeneous; many studies are retrospective, single-center, and small, with incompletely matched treatment regimens and inconsistent biospecimen timing. Cross-platform batch effects, variable preprocessing, and uneven availability of paired tissue, blood, imaging, and microbiome data hinder direct comparison across cohorts. These issues are most pronounced for HPD and irAE models, where event counts are low and unresolved confounding can exaggerate apparent performance. Consequently, many candidate biomarkers discussed in this review should be interpreted as biologically plausible and clinically promising, but not yet practice-defining, until prospective external validation and standardized implementation are achieved (64–66, 70).

Converging evidence indicates that multi-omics biomarkers capturing tumor, microenvironmental, and host determinants can provide a unified framework for estimating the likelihood of primary resistance, HPD, and immune-related toxicity during PD-1/PD-L1 therapy in lung cancer. This review supports a shift from isolated single-analyte markers toward rigorously validated composite signatures embedded in clinical workflows, with prospective trials required to confirm that such approaches enable rational patient selection, dynamic risk reassessment, and optimization of combination and sequencing strategies. If these conditions are met, multi-omics biomarkers are likely to become central to precision immuno-oncology in lung cancer, reducing catastrophic early failure and severe toxicity while maximizing the proportion of patients who derive durable benefit from PD-1/PD-L1 blockade.
